# 313. Host Protein Biomarkers Predicting Severity of Lung Damage due to COVID-19

**DOI:** 10.1093/ofid/ofab466.515

**Published:** 2021-12-04

**Authors:** Isabell Kerschbaumer, Melissa Fazzari, Shana R Burstein, Aisha Furey, Amy S Fox, Adam M Cole, Inessa Gendlina, Inessa Gendlina, Jacqueline M Achkar

**Affiliations:** 1 Albert Einstein College of Medicine, Bronx, New York; 2 Montefiore Medical Center/Albert Einstein College of Medicine, Bronx, New York

## Abstract

**Background:**

Biomarkers to predict the severity of lung damage due to COVID-19 are urgently needed to inform management and treatment decisions. Our objective was to investigate the predictive value of host proteins for worsening respiratory failure in one of the by COVID-19 most affected and diverse patient populations in the US.

**Methods:**

We performed a prospective single-center cross-sectional study of 34 adult patients admitted to Montefiore Medical Center in the Bronx, New York, for respiratory symptoms due to PCR-confirmed COVID-19. Exclusion criteria were age < 21, history of prior SARS-CoV-2 infection, and/or underlying severe chronic lung diseases requiring home O2 and/or high dose steroids. We stratified and compared patients by whether they developed worsening respiratory failure, necessitating transfer to the intensive care unit (ICU) during their hospital stay. Using a custom Luminex Assay, we measured hospital admission serum concentrations of 8 host proteins, representing respiratory-associated epithelial (RAGE, SP-D, CC16), endothelial (Ang-2, vWF), and immune pathways (S100A12, ICAM-1, VCAM-1).

**Results:**

Except for race and WHO COVID-19 scores, demographics, co-morbidities, symptoms, and symptom duration were not statistically significantly different between patients requiring transfer to the ICU (n=15) and non-ICU patients (n=19). Higher log-transformed levels for 5/8 proteins (S100A12, ICAM-1, Ang-2, RAGE, SP-D) showed significant or marginally significant increased cause-specific hazard for ICU transfer (n=15). Estimated cumulative incidence functions further showed a significantly or near significantly increased risk for ICU transfer for patients with above the median values of S100A12 or ICAM-1 (p=0.013), Ang-2 (p=0.056) or RAGE (p=0.077), respectively (Figure 1). Host proteins predicting need for ICU transfer did not correlate strongly with other clinical laboratory markers for COVID-19 severity (CRP, LDH, D-Dimer, Fibrinogen, Ferritin).

Figure 1. Patients with above median levels of host protein markers S100A12, ICAM-1, Ang-2, and RAGE have a significantly or near significantly increased risk for severe respiratory failure requiring transfer to the ICU.

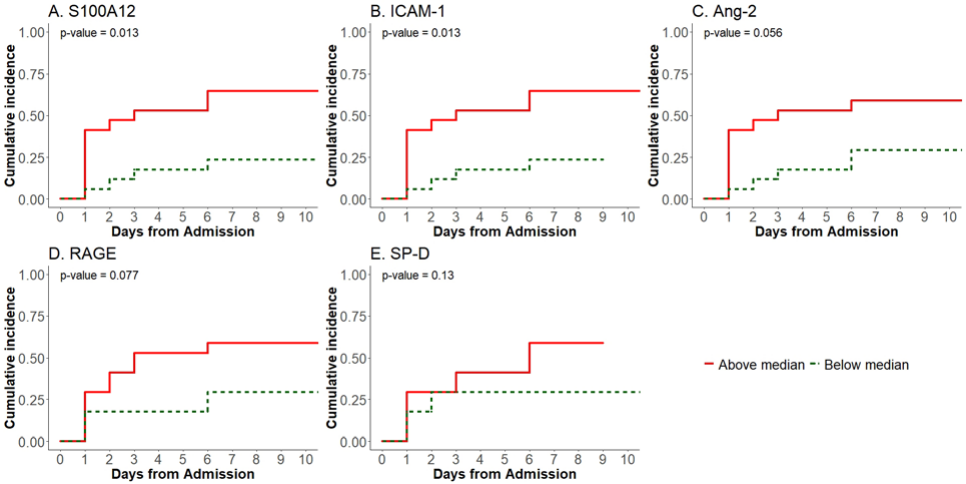

Comparison of estimated cumulative incidence at 7 days post admission for host protein markers above and below median levels for (A) S10012 (median 96,675 pg/ml); (B) ICAM-1 (median (1,192,277 pg/ml); (C) Ang-2 (median 3463 pg/ml); (D) RAGE (median 6356 pg/ml); and (E) SP-D (median 11,832 pg/ml).

**Conclusion:**

These results suggest that host proteins have additional predictive value for the severity of COVID-19-associated lung damage at time of presentation to the hospital.

**Disclosures:**

**Inessa Gendlina**, Nothing to disclose

